# Complete Genomic Sequence of Bacteriophage Felix O1^†^

**DOI:** 10.3390/v2030710

**Published:** 2010-03-09

**Authors:** Jean M. Whichard, Lee A. Weigt, Douglas J. Borris, Ling Ling Li, Qing Zhang, Vivek Kapur, F. William Pierson, Erika J. Lingohr, Yi-Min She, Andrew M. Kropinski, Nammalwar Sriranganathan

**Affiliations:** 1 Mailstop G29, Centers for Disease Control and Prevention; 1600 Clifton Road, Atlanta, GA 30329, USA; E-Mail: zyr3@cdc.gov (J.M.W.); 2 Smithsonian National Institution, National Museum in Natural History, MRC 534, Washington, DC 20560, USA; E-Mail: weigtl@si.edu (L.A.W.); 3 Abbot Point of Care, 185 Corkstown Road, Ottawa, ON, K2H 8V4, Canada; 4 Pennsylvania State University, Department of Veterinary and Biomedical Sciences, 204 Wartick Laboratory, University Park, PA 16802, USA; E-Mail: lul17@psu.edu (L.L.L.); 5 Fred Hutchinson Cancer Research Center, 1100 Fairview Ave. N. Seattle, WA 98109, USA; E-Mail: qing@fhcrc.org (Q.Z.); 6 Pennsylvania State University, 115 Henning Bldg., University Park, PA 16802, USA; E-Mail: vkapur@psu.edu (V.K.); 7 VA-MD Regional College of Veterinary Medicine, Virginia Polytechnic Institute and State University, Duck Pond Drive (0442), Blacksburg, Virginia 24061, USA, E-Mail: pierson@vt.edu (F.W.P.); 8 Public Health Agency of Canada, Laboratory for Foodborne Zoonoses, Guelph, Ontario N1G 3W4, Canada; E-Mails: Erika_Lingohr@phac-aspc.gc.ca (E.J.L.); kropinsk@queensu.ca (A.M.K.); 9 Centre for Biologics Research, Health Canada, Room D159, Frederick G. Banting Building 251 Sir Frederick Banting Driveway, Tunney’s Pasture, Ottawa, ON K1A 0K9, Canada; E-Mail: yi-min.she@hc-sc.gc.ca (Y.-M.S.); 10 University of Guelph, Department of Molecular and Cellular Biology, Guelph, Ontario N1G 2W1, Canada; 11 Center for Molecular Medicine and Infectious Disease; 1410 Prices Fork Road; Blacksburg, VA 24061-0342, USA

**Keywords:** *Salmonella*, bacteriophage, *Myoviridae*, Felix O1, DNA sequence, bioinformatics

## Abstract

Bacteriophage O1 is a *Myoviridae* A1 group member used historically for identifying *Salmonella*. Sequencing revealed a single, linear, 86,155-base-pair genome with 39% average G+C content, 131 open reading frames, and 22 tRNAs. Closest protein homologs occur in *Erwinia amylovora* phage φEa21-4 and *Escherichia coli* phage wV8. Proteomic analysis indentified structural proteins: Gp23*,* Gp*36* (major tail protein)*,* Gp*49,* Gp*53,* Gp*54,* Gp*55,* Gp*57,* Gp*58* (major capsid protein)*,* Gp*59,* Gp*63,* Gp*64,* Gp*67,* Gp*68,* Gp*69,* Gp*73,* Gp*74* and Gp*77* (tail fiber). Based on phage-host codon differences, 7 tRNAs could affect translation rate during infection. Introns, holin-lysin cassettes, bacterial toxin homologs and host RNA polymerase-modifying genes were absent.

## 1. Introduction

Among the large tailed viruses of the order *Caudovirales* [1] infecting *Salmonella* are 44 members of the *Myoviridae* (phages with contractile tails), 68 members of the *Siphoviridae* (viruses with long noncontractile tails) and 64 incidences of *Podoviridae* (short noncontractile tails). In addition to the phages described in 2007 [2,3] a considerable number of *Salmonella* phages and prophages have been recently reannotated, sequenced or described in publications. These include: ɛ34 [4], φSG-JL2 [5], c341 (NC_013059), E1 [6], Fels-1 (NC_010391), Fels-2 (NC_010463), Gifsy-1 (NC_010392), and Gifsy-2 (NC_010393), KS5 (NC_006940), SE1 (NC_011802), SETP3 (NC_009232). Of all the sequenced phages only KS7 and SP6, members of the *Podoviridae* and, E1, KS5 and SETP3 (*Siphoviridae*) are lytic.

Bacteriophage O1 (also called phage Felix O1, 01 or 0–1) is a member of the A1 group of the *Myoviridae* [1,7] with an icosahedral head 73 nm in diameter and a contractile tail (17 x 113 nm) terminating in six straight tail fibres. Its morphology is such that it is immediately recognizable in the EM. It was discovered in England and first used in 1943 by Felix and Callow in the original scheme for the identification and typing of *Salmonella* Typhi [8]. Phage O1 is fairly unique among *Salmonella* bacteriophages because of its relative *Salmonella*-specificity. The phage lyses 98.2% % of *Salmonella* and less than 1.4% of other *Enterobacteriaceae* [9]. Of 15 serogroups tested in another study, all but two included isolates that could be productively infected by phage O1 [10,11]. Kallings also observed that other Gram-negative enteric bacteria are generally resistant to lysis by this phage (see references in [7]). The somatic receptor for phage Felix O1 is lipopolysaccharide [12,13]. Since this phage infects almost all *Salmonella* isolates, it has been proposed as a therapeutic or /decontaminating agent [14], and has used as a diagnostic reagent [15,16]. A derivative of Felix O1 carrying the *luxAB* genes has been constructed to detect *Salmonella* bacteria in food samples [17,18].

The three most important *Salmonella* phages are P22, because of its roles in transductional analysis and morphogenesis, ɛ15, because of its role in understanding lysogenic conversion of serotype, and Felix O1. The sequence of this bacterial virus was completed in 2000, and we present a detailed analysis of its genome, proteome and transcriptome.

## 2. Results and Discussio

### 2.1. Genome Assembly and DNA Sequence

The 1,493 sequencing reactions were carried out resulting in >7–fold coverage. The individual sequences were assembled into a single contiguous sequence of 86,155 bp. Since pulsed-field gel electrophoresis indicated that the phage genome is a single double-stranded linear DNA molecule of approximately 86.5 kb the sequence data suggests that this phage possesses short terminal repeats. As with other phages containing RIIAB-encoding genes, for annotation purposes, the Felix O1 genome was opened just upstream of the *rII*A homolog. The genome has an average mol% G+C content of 39.0% which is significantly lower than that of their hosts (*ca.* 50% GC). It has been our observation that the lytic phages vary more than the temperate ones in the disparity between their base composition with that of their hosts. This will be discussed in greater depth in the section on tRNAs. A consequence of its low G+C content is its natural resistance to many *Salmonella* restriction enzymes (*Sba*I, *Sbl*, *Spt*AIP, *Sen*, *Spo*I, *Ssh*AI, *Sth*, *Sty*I) [19] which recognize GC-rich sequences. Lastly, the genome contains twelve direct repeats of ≥ 24 bp. Two 57 bp direct repeats (CTTTACATGGGGCTTAAAAGTCTGTAAAGTAAGCCCCAGATAAAGAGCTTTACCACT) are located between genes *15* and *16,* and between *19* and *20*; 39 bp repeats (TTGACACTGGTTTTTAGATAGATTA AATTACACATCAAC) are located between genes *18* and *19,* and between *20* and *21*; and, *51* (GTCTCAGGGACTGAACAGGTTTCCAGTGTAGCTGGTGACCACTCACA CACT), and 32 bp (ACTGGTGCTCACACCCACTCAGTGAGTGGTTC and GGTGACTCTATCGGTGGTAAACATCGTG TTCA) are found in genes *76* and *77.* The latter nucleotide repeats result in regions of amino acid sequence similarity shared between gp*76* and gp*77*.

### 2.2. Identification and Analysis of Open Reading Frames (ORFs)

**Figure 1:**
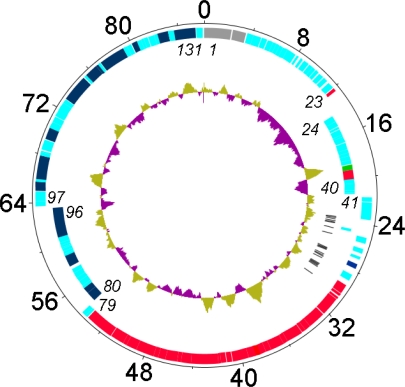
Genetic and physical map of phage Felix O1 prepared using DNAPlotter [20] with, from outer to inner rings, the size in kb; genes on the forward and reverse strands with some of the genes listed in italics (N.B. gene *1* = *rIIA*), and tRNA-encoding genes (black). The inner circles correspond to a GC plot (purple, below average GC-content; greenish-brown) and a GC skew analysis. Genes involved in nucleotide metabolism or DNA replication are indicated in dark blue, those involved in DNA packaging and morphogenesis in red; and, the lysis gene in dark green.

The genome was reanalyzed using Kodon coupled with Psi-BLAST analyses to reveal the 131 ORFs which are described in this manuscript (Figure 1; Supplementary Figure 1, Supplementary Table 1). As with other phage genomes a high percentage of the genome is devoted to genes (90.6% ORFs, 1.8% tRNAs). The number of predicted ORFs per kb of sequence is 1.5 which corresponds well to the values for other bacteriophages including T4 (1.7), T7 (1.4), and T5 (1.3). By comparison the gene density of *S*. Typhimurium LT2 is 0.9. Again, as is commonly observed, relatively few of the genes possessed homologs when the data were originally deposited in GenBank.

**Figure 2:**
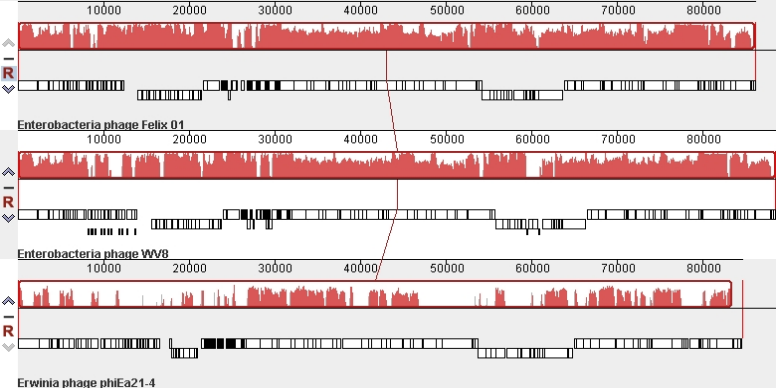
Mauve alignment of the genomes of phages Felix O1 (top), wV8 (middle) and φEa21-4 (bottom). The degree of DNA sequence similarity is indicated by the height of the red-coloured regions. Just above the phage names are box-like diagrams indicating the position of the genes.

The recent completion of the *Erwinia amylovora* φEa21-4 [21] and *Escherichia coli* wV8 [22] phage genome sequences revealed that these two members of the *Myoviridae* possessed numerous Felix O1 homologs. As shown using CoreGenes [23,24], φEa21-4 shares 69 homologs while wV8 has 121. A comparison of these viruses at the DNA level using Mauve [25,23] reveals that while Felix O1 and wV8 share very similar genome organization and sequence similarity, the sequence similarity to φEa21-4 is mainly found between 27–46, and 61–86 kb (Figure 2). These correspond to regions involved in morphogenesis and DNA replication/nucleotide metabolism, respectively.

Based upon the close similarity between these three phages Lavigne *et al.* [26] have proposed the creation of a new genus, the “FelixO1-like viruses” to encompass them. In the following paragraphs we will discuss the homologs with respect to nucleotide metabolism, DNA replication, phage morphogenesis and lysis.

### 2.3. Nucleotide Metabolism and DNA Replication

Felix O1 contains numerous genes involved in nucleotide metabolism and DNA replication. The former comprise an exodeoxyribonuclease, the anaerobic and aerobic ribonucleotide reductase subunits, ribose phosphate pyrophosphokinase, nicotinate phosphoribosyltransferase, dihydrofolate reductase, deoxynucleoside monophosphate kinase and thymidylate synthase. Among the enzymes involved in DNA replication a DNA ligase, DNA polymerase, and primase/helicase were identified. Phosphoribosyl pyrophosphate synthetase (PRPP synthetase, EC 2.7.6.1) forms 5-phospho-α-D-ribose 1-diphosphate which is an early intermediate in histidine biosynthesis, as well as an intermediate in purine and pyrimidine syntheses [27]. PRPP is also a precursor in the formation of nicotinate nucleotide from quinolinate ultimately contributing to the biosynthesis of NAD. The aerobic and anaerobic ribonucleoside-diphosphate reductases are responsible for the interconversion of ribo- to deoxyribonucleotides. In this pathway the reducing activity derives from thioredoxin or glutaredoxin. Since the Felix O1 *nrdABDG* cluster includes an 80-amino acid protein with sequence similarity to glutaredoxin, it is likely that the phage Nrd homologs employ this reducing agent rather than thioredoxin. Dihydrofolate reductase (EC 1.5.1.3) reduces 7,8-dihydrofolate to tetrahydrofolate which plays a role in RNA and DNA precursor syntheses and as a cofactor in the conversion of dUMP to dTMP by thymidylate synthase (EC 2.1.1.45) [28]. In bacteria, four enzymes are involved in the conversion of dNMPs to their dinucleotide counterparts. In phage-infected cells this is often accomplished by a single broad-substrate specific enzyme deoxynucleotide kinase (possibly Felix O1gp*99*).

We might expect that Felix O1, like other virulent members of the *Myoviridae*, would hydrolyze the host genome and reutilize the deoxyribonucleotides in progeny DNA biosynthesis. No genes specifying deoxyribonuclease homologs were identified, but Felix O1 does produce a putative 5’-exonuclease (gene *105*). Gp*88* contains a full-length pfam01068 (DNA_ligase_A_M) ATP-dependent DNA ligase domain. Interestingly, the closest homologs, after phages wV8 and φEa21-4, are among eukaryotic ligases including those of *Trypanosoma, Leishmania* and *Crithidia*. The 906 amino acid Felix O1 DNA polymerase (Gp*96*) shows greatest overall sequence similarity to the DNA polymerases of members of the *Podoviridae: P. aeruginosa* phage PaP3 [2e-24; NP_775225], *Salmonella* phage SP6 [8e-21;NP_853574] [29], and coliphages K1E [8e-12; YP_424986], K1-5 [3e-12; AAR90053] [30] and T7 [6e-09; NP_041982] [31]. Gp*101* contained a cd01122 motif (GP4d_helicase) described as a homodimeric 5’-3’ helicase. Its homologs are to primase/helicases in T7-like members of the *Podoviridae: Morganella* phage MmP1 [2e-49; YP_002048644], *Yersinia* phage Berlin [4e-46; YP_918996] and enterobacterial phage BA14 [1e-43; YP_002003469].

### 2.4. Codon Usage and tRNAs

A comparison of the codon usage pattern of the phage and its potential hosts reveals some interesting differences (Supplementary Table 2). Emphasizing the codons which are used frequently (≥30% of incidences) and show a ≥1.5 fold increase in use we can identify 13 tRNA codons which are significantly overrepresented in the phage genes (Gly[GGT], Val]GTA], Val[GTT], Ala[GCA], Ala[GCT], Arg[AGA], Ser[TCT], Lys[AAG], Thr[ACA], Thr[ACT], Gln[CAA], Pro[CCA] and Pro[CCT]). The amino acid utilization patterns of the phage proteins and, as a comparator, *S*. Choleraesuis, are also shown in this table. The only amino acids which are significantly overrepresented in the phage proteome were serine, lysine, asparagine and cysteine.

The GC content of the first, second and third codon positions are 45, 39 and 33% for Felix O1, while the average of the four *Salmonella* serotypes is 59, 41 and 59%. We might therefore expect that effective translation of this phage’s mRNAs may be compromised unless it encodes compensatory tRNAs and that initiation codon usage may also differ from than the host. The average initiation codon use for the four *Salmonella* serotypes is ATG (80.1%), GTG (11.6%) and TTG (7.8%) [32]. In contrast, and possibly because of the higher AT content of its genome, 94.7% phage ORFs begin with ATG, 3.0% with GTG, and in 2.3% of cases TTG is the initiation codon.

An analysis employing tRNAscan-SE [33] revealed 22 putative tRNAs, two of which, identified as numbers 5 and 20 in Table 1, are described as pseudo tRNA genes. The latter have also been documented in mycobacteriophage Bxz1 (NC_004687), cyanophage S-PM2 (NC_006820), and *Lactobacillus* phage LP65 [34] and represent molecules which lack conserved structural or sequential features or, contain insertions or deletions [33]. The tRNAs were clustered into three groups of eight, eight, and five genes respectively followed by a single tRNA 851 nt downstream (bases 30,105 to 30,180). Transfer RNA genes are common among myoviruses with large genomes but only four of the 48 fully sequenced members of the *Myoviridae* with genomes less than 100 kb have tRNA genes: φP27 (NC_003356), BcepNY3 (NC_009604), P1 (NC_005856) and φEcoM-GJ1 (NC_010106).

In several incidences (Ala[GCA], Val[GTA], Lys[AAG], Thr[ACA], Gln[CAA], Leu[TTA] and Pro[CCA]) the presence of phage encoded tRNAs presumably has a positive impact of translation. Interestingly one might have expected tRNAs for the following highly represented codons Gly[GGT], Val[GTT], Arg[AGA], Ser[TCT], Thr[ACT] and Pro[CCT]. The presence of a Met tRNA in Felix O1 and many other phages suggests that the augmentation by this tRNA may play a positive role in translational initiation in phage-infected cells.

### 2.5. Transcription

Based upon the assumption that the genome of Felix O1 becomes circular during intracellular replication, its genome displays four transcriptons, that is, regions of transcription. These involve genes *97–131/1–23*, *96–80*, *40–24*, and *41–79*. Since DNA replication is an early function in phage-infected bacteria we assume that transcription is initiated from divergent promoters located between genes *96* and *97*. The putative morphogenesis and packaging genes are part of gp*41–79* transcriptional unit which presumably occurs later in the infective cycle. It is odd therefore that the divergent transcription occurs between genes *40* and *41*. One would assume that transcription of the tRNA gene clusters would also occur early as they do in T4.

Unlike members of the T7 family of phages, Felix O1 does not code for its own RNA polymerase (RNP), nor does it apparently encode, as does T4, for proteins which modify host RNA polymerase promoter specificity. Furthermore, we were able to identify several potential promoters bearing significant sequence similarity to those recognized by host RNP upstream of both putative early and late genes: P*11* (TTGACA(N13)TGATTTATA 6658–6683), P*41* (TTGACA(N15)TATAGG 21515–21541), P*45* (TTGACA(17)TATAGT 25600–25628), P*102* (TTGACC(N17)TATACT 67839–67867) and P*117* (TTGAAA (N19)TATAAC 77026–77056). Other than P*41* no other obviously homologous sequences were discovered in the *40–41* or *96–97* intergenic regions. How transcription around the numerous rho-independent terminators (Table 2) is accomplished, either by read-through or the presence of down-stream secondary promoters is not known, nor is how transcription is temporally regulated in this bacteriophage.

Of the 102 predicted ORFs analyzed by RT-PCR, all produced a transcript associated with early, middle or late stage infection of susceptible *Salmonella* Typhi (Table 3, Supplementary Table 3). The 102 predicted ORFs that were tested included ORFs associated with nucleotide metabolism, DNA replication, transcription regulation, packaging, morphogenesis, and lysis. In many cases, timing of ORF transcription was as expected based on predicted function (Table 3). For example, ORFs *96, 101*, and *105*, involve in DNA metabolism and replication, were transcribed early (within 5 minutes of host infection). ORFs predicted to be involved in packaging and morphogenesis (ORFs *72, 73, 76* and *77*) were transcribed late (60 minutes after infection).

### 2.6. Packaging and Morphogenesis

Unlike most phages which have a cassette composed of the large and small terminase subunits (TerS & TerL), Felix O1 appears only to have a TerL homolog (gp*52*). Other phages which apparently share this trait include LP65 [34], *Listeria* phage P100 [35], and *Streptococcus pneumoniae* temperate phage EJ-1 [36]. Interestingly, after homology to the single terminases of phages wV8, φEa21-4 and coliphage rV5 (2e-64, YP_002003567) the closest Felix O1 TerL homologs are often found in the domain Archaea. They include *Methanosarcina acetivorans* C2A terminase [3e-70, NP_618697], the large terminase subunit from *Methanobacterium* phage ψM2 [2e-47, NP_046964], and the putative large terminase subunit from *Methanothermobacter wolfeii* prophage ψM100 [1e-46, NP_071818].

While a large number of the putative morphogenesis genes (Gp*53–77*) possess homologs to hypothetical phage or prophage gene products only four were initially identified through homology searches: gp*56* (head maturation protease) gp*71* (base plate protein), gp*76* (tail fiber) and gp*77* (tail fiber).

Gp*56* was identified as possessing pfam01343 [Peptidase_S49] and COG0616 [SppA, periplasmic serine proteases (ClpP class)] motifs. Its homologs include phage *Klebsiella oxytoca* linear prophage φK02 head-maturation protease [4e-30, YP_006585] [37], and prohead protease ClpP from *Burkholderia cepacia* phage BcepNazgul [6e-30, NP_918994]. The presence of a gene specifying a head maturation protein suggests that the major head protein is proteolytically cleaved during capsid morphogenesis as are many phage capsid proteins. Alternatively, this protein maybe involved in hydrolysis of a scaffolding protein.

Because of its mass and position relative to the *terL* (gene *52*) gene *53* might encode the portal protein. Gp*53* contains a pfam06074 [protein of unknown function (DUF935)] motif which is conserved in phage Mu gp*29*. Repeated Psi-Blast iterations confirmed its relationship to coliphage Mu gp*29* [NP_050633] and its homologs in *Haemophilus influenzae* (FluMu [VG29_HAEIN]), *H. ducreyi* [NP_872736] and *P. aeruginosa* phage D3112 [NP_938234].

Again based upon its mass and position relative to the putative head maturation protease we might suggest that Gp*58* is the major head protein for this bacteriophage. Reiterated Blast analyses showed its general sequence relatedness to a wide variety of “conserved hypothetical proteins.” It also shows sequence similarity to gp*20* of *Wolbachia* bacteriophage WO [BAA89645].

Gp*71* is related to phage P2 baseplate assembly protein gpV and its homologs are encountered in several *E. coli* strains along with the putative baseplate protein of *Aggregatibacter* phage S1249 [2e-11, YP_003344788].

The product of gene *76* shows sequence similarity, at its C-terminus, to a variety of prophage or phage proteins, including T4 gp*37* [3e-14, NP_049863] which is the distal subunit of the long tail fibre protein. Gp*77* also shows homology to this protein at its C-terminus but multiple regions of sequence relatedness to phage tail fiber proteins are found in gp*H* of *Yersinia* phage L-413C [NP-839867] and gp*36* - the hinge connector of long tail fiber distal connector of coliphage 44RR2.8t [NP_932576] and of *Aeromonas* phage 31 [YP_238947]. Interestingly, another gene (*36*) identified as being a tail protein was identified in a region well separated from the bulk of the morphogenesis genes.

### 2.7. The Felix O1 Proteome

**Figure 3:**
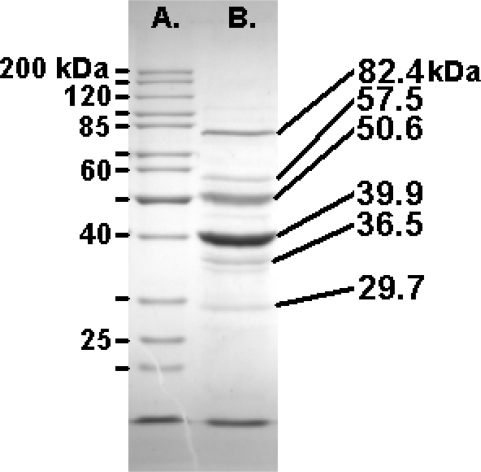
SDS-PAGE (10%) analysis of the structural proteins of Felix O1 with the masses of the Fermentas PageRuler markers (left) and the most abundant proteins (right).

Felix O1 purified in a CsCl equilibrium gradient was analyzed by denaturing polyacrylamide gel electrophoresis [38] revealing ten visible bands ranging from 25 - 110 kDa. The three major high molecular weight protein bands were 82.4, 50.6, and 39.9 kDa (Figure 3).

Trypsin digestion followed by mass spectrometry revealed that the 82 kDa protein is encoded by gp*77* (38% coverage; MS-Fit MOWSE score 3.4e^10^) while the 51 kDa viral protein is specified by gp*49* (27% coverage; score: 6.6e^6^). In both of these cases peptides were recovered from the N-terminus suggesting absence of post-translational cleavage. The 40 kDa protein, translated from gene *58* (39% coverage; MOWSE: 1.5e^10^) was only represented by peptides starting at residue 78. While no evidence was found for the peptides corresponding to amino acids 10–36 and 37–62 the observed mass of the protein (40 kDa) is remarkable similar to the predicted size (41.5 kDa). This suggests that this protein is probably not modified by Gp*56* but that the latter is involved in removal of the scaffold. While it is tempting to speculate, based upon synteny and mass, that Gp*49* is the sheath protein of this phage we have no evidence for this based upon homologs or the extensive use of motif scanning using MEME [39].

In addition, a whole-phage shotgun analysis (WSA; [40,41]) was applied with phage samples. Using very stringent identification criteria the products of the following ORFs were identified as phage structural protein: 23*, 36, 53, 54, 55, 57, 58, 59, 63, 64, 67, 68, 69, 73, 74* and 77 (Supplementary Figure 2; Table 4A and 4B). Furthermore, since the major coat protein (Gp58) N-terminal peptide fragment was identified, proteolytic processing can be eliminated from consideration.

### 2.8. Lysis

In most tailed phages, a gene cassette containing lysin and holin components are found in the genomic sequence. As with coliphage T4, a holin gene is not adjacent to the putative lysin (gp*35*). Lysins are small proteins usually have two or three transmembrane domains, a charged C-termini and display little sequence homology with other similarly functioning proteins. Based upon these limited criteria, any one of gp*79*, gp*116*, gp*118*, gp*119*, or gp*131* of Felix O1 could be the holin. The lysin (gp*35*) contains a high scoring cd00737 (endolysin_autolysin) and pfam00959 (Phage_lysozyme) motifs. Among bacteriophages, the closest homologs are found in *Pseudomonas* phage proteins [phage PA11, 6e-23, YP_001294626; and, phage PAJU2, 2e-22, YP_002284361], and *Acyrthosiphon pisum* bacteriophage APSE-1 P13 protein [2e-22, NP_050974].

### 2.9. Introns and Homing Endonucleases

Many phages with large genomes have introns. T4 has type I introns in the *nrdB, nrdD* and *td* (thymidylate synthase) genes [42]. *Bacillus* phage SPβ also has introns in two of its ribonucleotide reductase genes [43]. Two other examples are *Synechococcus* phage S-PM2 [44] and *Staphylococcus* phage K [45]. In the former an intron is found in a gene (*psbA*) which specifies a protein in the photosynthetic reaction center, while in the latter, there are introns in the DNA polymerase gene and another in the lysin gene. Felix O1 does not contain insertions within any of these genes. On the other hand its genome contains six copies of sequences homologous to homing endonuclease (HNH endonucleases). This high number of HNH endonucleases has only been found in *Xanthomonas oryzae* phage Xp10, where the authors considered that they played a significant role in the evolution of that siphoviral genome [46]. Complete or potentially defective homing endonucleases can be found as individual ORFs, within introns or as inteins (e.g., in-frame intervening sequences). The latter are commonly found associated with introns. The question arises whether the HNH endonuclease genes are associated, in Felix O1 with type I introns or with inteins. None of the closely juxtaposed genes to the homing endonuclease were in the same reading frame as the flanking sequences ruling out the possibility of inteins. This does not rule out the possibility of introns in the Felix O1 chromosome, but if introns are present, they apparently do not reside in the same genes that contain them in T4 or are not homologous with those found in T4.

## 3. Experimental Section

### 3.1. Phage and Bacterial Strains

Phage Felix O1 was kindly provided by Dr. Hans-W. Ackermann (Felix D’Herelle Bacteriophage Stock Center, Université Laval, Quebec, Canada). Dr. Ackermann also provided the host strain of *Salmonella enterica* subsp. *enterica* serovar Typhi (ViA, phage type Tananarive) used to propagate the phage. Bacterial stock cultures were stored at −80°C in 20% glycerol while bacteriophage stock cultures were stored at −80°C in low salt buffer (0.05M Tris HCl, pH 7.5, 0.05M NaCl, 0.01M MgSO_4_) until used.

### 3.2. Isolation of Felix O1 DNA

Phage Felix O1 was propagated using the plate lysis technique [47] with host and phage incubated on 150-mm Difco Trypticase soy agar plates supplemented with 0.5 mM CaCl_2_. After overnight incubation at 37 °C, the plates were flooded with 9 ml of suspension buffer (0.05 M Tris HCl, pH 7.5, 0.1 M NaCl, 8.1 mM MgSO_4_). The surfaces of to the plates were scraped using a glass spreader, and the resulting liquid was placed on ice. Following 8–10 min treatment with chloroform at room temperature the residual bacterial debris was removed by centrifuging at 13,700 x g for 10 min. at 4 °C. DNA was extracted directly from this crude lysate using a Qiagen® Lambda kit (Qiagen Inc., Valencia, CA) for use in the preparation of a randomly-sheared library, and for direct genomic and PCR primer walking.

### 3.3. Sequencing Strategy

A randomly-sheared genomic library was made according to the methods of Roe (http://www.genome.ou.edu/protocol_book/protocol_partII.html). Plasmids containing phage inserts were sequenced using T3 and T7 primers on an ABI 377 automated sequencer with BigDye Terminator chemistry at the Advanced Center for Genetic Analysis (University of Minnesota, St. Paul, MN). Gap closure was achieved by primer walking on phage DNA or PCR amplification and sequencing of the amplicons at the Virginia Tech Sequencing Facility. ABI Editview software (Foster City, CA) and Sequencher (Gene Codes Corporation, Ann Arbor, MI) were used to align raw sequence data and to produce the Felix O1 chromosome consensus.

### 3.4. RT-PCR Methods

RT-PCR was used to determine mRNA expression [48]. *Salmonella* Typhi was grown to log phase in 80 ml TSB. Bacteria were pelleted, and resuspended to approximately 1 x 10^9^ cfu/ml. Two-milliliter aliquots of Felix O1 phage in low salt buffer (1 x 10^10^ pfu/mL) were used to infect 2-mL aliquots of the resuspended culture for 0, 5, 10 or 25 min at which time they were interrupted by placing in a dry ice ethanol bath, and lyophilized overnight. Nucleic acid was isolated the following day using a Micro to Midi Total RNA Purification® kit (Invitrogen) according to manufacturer’s instructions. TRIzol® reagent (Invitrogen) was used according to manufacturer’s instructions to further purify RNA, and residual DNA was inactivated with DNase I (100 units per aliquot; Sigma Aldrich, St. Louis, MO) at 37°C for 2 h. A sample of Felix O1 phage was also processed to confirm absence of mRNA in the phage stock.

RT-PCR primers were designed using Lasergene® software (DNASTAR, Inc.; Madison, WI) based on putative ORFs, and RT-PCR reactions were performed using Ready-To-Go™ RT-PCR Beads (GE Healthcare) according to manufacturer’s instructions; 20 pmol of each primer and 1 μl of total RNA were included in each reaction. Primers used are shown in Supplementary Table 3. Amplicons were resolved in 1.5% agarose gels and were visualized using ethidium bromide.

### 3.5. DNA Sequence Analysis

The GC content and presence of direct repeats was analyzed using DNAMAN (Lynnon Corp., Vaudreuil-Dorion, QC, Canada) while skew analyses was carried out with Jie Zheng’s DNA base composition analysis tool (http://molbiol-tools.ca/Jie_Zheng/).

Open reading frames were identified Kodon (Applied Maths, Inc., Austin, TX) with each of the putative proteins screened against the GenBank nonredundant protein database (http://www.ncbi.nlm.nih.gov) using Psi-BLAST [49] and Batch BLAST (http://greengene.uml.edu/programs/NCBI_Blast.html). Proteins showing homology were compared using GENESTREAM’s ALIGN program at http://xylian.igh.cnrs.fr/bin/align-quess.cgi. In addition, the proteins were scanned for transmembrane domains using the TMHMM program (http://www.cbs.dtu.dk/services/TMHMM-2.0/).

The genomic sequence was examined for genes coding for transfer ribonucleic acids using tRNAscan-SE software (http://lowelab.ucsc.edu/tRNAscan-SE/) [33]. Codon usage information for *Salmonella* serotypes Choleraesuis, Paratyphi A, Typhi and Typhimurium was generated from the data in the Codon Usage Database [50] (http://www.kazusa.or.jp/codon/). The codon usage for Felix O1 was determined using Paul Stothard’s “The Sequence Manipulation Suite” at http://www.bioinformatics.org/SMS/. Transcriptional terminators were identified using TransTerm [51] and RibEx:Riboswitch Explorer [52]. Potential promoters were located using Martin Reese’s Neural Network Promoter Prediction (http://www.fruitfly.org/seq_tools/promoter.html). Only sites located in intergenic regions were considered.

### 3.6. Proteomics of Felix O1

Felix O1 was propagated in Luria broth at 37 °C using *S*. Typhimurium LT2 as the host. The phage was precipitated from the clarified lysate using polyethylene glycol 8000 and banded twice in CsCl equilibrium gradients [53]. The phage band was dialyzed against TE buffer and lysed using Laemmli sample buffer [38]. After boiling for 5 min. the samples were sonicated to decrease the viscosity of viral DNA. The structural proteins were separated on 10 and 12.5% gels of polyacrylamide in the presence of SDS, and stained using SimplyBlue SafeStain (Invitrogen Corporation, Carlsbad, CA). The data was analyzed using BioNumerics (Applied Maths). The three most intense phage bands were excised and subjected to in-gel digestion with trypsin at the University of Guelph Biological Mass Spectrometry Facility (Guelph, ON, Canada). The peptides were resolved and sized using a Reflex III MADI-TOF (Bruker Daltonics Inc., Billerica, MA). The masses of the tryptic peptides were determined using Genomic Solutions (Ann Arbor, MI) Prowl program ProFound against the NCBI nonredundant protein database, and against a custom Felix O1 protein database using Protein Prospector’s MS-FiT [54].

In addition a whole-phage shotgun analysis (WSA; [40,41]) was applied to phage samples reduced with DTT and alkylated with iodoacetamide, then digested with trypsin. LC MS/MS analysis was conducted by a 90 min. gradient of solvent A (0.1 formic acid) and solvent B(99.9% acetonitrile/0.1% formic acid) on a Thermo LTQ-FT instrument coupled with Waters UPLC. The MS measurements on the FT instrument have a high mass accuracy of 1–2 ppm, and the proteins were identified at a false positive rate less than 0.65%.

### 3.7. Genome Sequence Accession Number

The annotated genomic sequence for this phage is available from NCBI under accession numbers AF320576 and NC_005282.

## 4. Conclusions

Along with phages P22 and ɛ15, Felix O1 is the most important *Salmonella* virus, and the only member of this triumvirate which is lytic. In this manuscript we have completely updated the analysis of its genome conducted in 2000 (AF320576). In addition, we have presented a detailed investigation of its proteome and preliminary transcriptomic analysis.

The use of two complementary approaches to identifying the structural proteome – characterization of individual bands excised from polyacrylamide gels, and the WSA approach – permitted accurate identification of far more proteins than either technique separately. We have positively identified the following as structural proteins: Gp23*,* Gp*36* (major tail protein)*,* Gp*49,* Gp*53,* Gp*54,* Gp*55,* Gp*57,* Gp*58* (major capsid protein)*,* Gp*59,* Gp*63,* Gp*64,* Gp*67,* Gp*68,* Gp*69,* Gp*73,* Gp*74* and Gp*77* (tail fiber). Because of its mass, and by comparison with the organization of other phage morphogenesis genes Gp53 may be the portal protein. Gp67 may function as the tail tape measure protein but further analyses will be required to verify these suppositions.

Based upon their phenotypic and genotypic characteristics, the International Committee on the Taxonomy of Viruses classifies all viruses into a series of genera, for example the “P22-like viruses.” Because sequenced phage genomes will always be in the minority relative to the global phage genome pool, this has led to the creation of weak or false relationships which require reexamination as new data enters GenBank. Two examples of this were Roseophage SIO1 [55] and *Pseudomonas aeruginosa* phage PaP3 (NC_004466) which were originally classified as a member of the “T7-like phages” but, with that phage, they only shared four and two homologs, respectively [24]. They were then placed with cyanophage P60 (NC_003390) into the taxonomically undefined “Cyanophage P60 group.” Unfortunately, they were equally unrelated to that phage: because they share only six and three homologs, respectively. This problem also held true for orphans such as Felix O1, T1, φKMV, and ɛ15 which when originally sequenced were only distantly related to known phages. We now recognize that close homologs exists: coliphage wV8 [22] and *Erwinia* phage φEa21-4 [21] in the case of Felix O1; Rtp [56] and TLS [57] with T1, LKD16 [58] for φKMV and φV10 [59] for ɛ15. These relationships were shown using CoreGenes [24], and exploited by Lavigne et al [60] to create a rational taxonomy of the *Podoviridae* based upon protein homology. We have recommend that the ICTV recognize *Salmonella* phage Felix O1 as the type species of a new genus, the “FelixO1-like viruses” [26]. This genus would contain Felix O1, *E. coli* phage wV8 [22] and *Erwinia* phage φEa21-4 [21].

## Supplementary Materials



## Figures and Tables

**Table 1:** Location of the tRNA genes in the Felix O1 genome, their cognate amino acids and anticodons.

**tRNA #**	**tRNA beginning**	**tRNA end**	**Amino acid**	**Anticodon**	**Codon**	**Cove Score**
1	23699	23775	Pro	TGG	CCA	68.25
2	23783	23860	Glu	TTC	GAA	62.48
3	23952	24028	Met	CAT	ATG	28.90
4	24112	24188	Asn	GTT	AAC	38.74
5	24258	24345	Pseudo (Tyr)	GTA	TAC	39.92
6	24351	24427	Asp	GTC	GAC	61.37
7	24856	24931	Lys	TTT	AAA	60.20
8	25509	25584	Ile	GAT	ATC	49.05
9	26975	27052	Leu	TAG	CTA	52.24
10	27060	27135	Lys(2)	CTT	AAG	59.65
11	27142	27217	Ala	TGC	GCA	38.73
12	27224	27298	Gly	TCC	GGA	56.50
13	27728	27804	Thr	TGT	ACA	61.15
14	27900	27974	Val	TAC	GTA	51.84
15	27976	28053	Leu(2)	CAA	TTG	46.15
16	28168	28243	Arg	ACG	CGT	63.45
17	28828	28903	Gln	TTG	CAA	47.31
18	28906	28984	Leu(3)	TAA	TTA	57.88
19	28990	29065	Gln(2)	CTG	CAG	50.21
20	29097	29172	Pseudo (His)	GTG	CAC	42.31
21	29179	29254	Phe	GAA	TTC	43.77
22	30105	30180	Cys	GCA	TGC	47.75

**Table 2:** Rho-independent terminators in the genome of Felix O1 predominantly discovered using TransTerm, with the structures verified using MFOLD.

**Name**	**Position**	**Sequence**	**dG** (- kcal/mol)
t*2*	3499..3531	ggctgcttcggcggccttttttatttgtatttt	14.00
t*5*	5291..5316	ggctccttcgggagcctttttcattt	14.90
t*13*	7947..7994	gcctttcttatctggtaaatttttcaggtaaggagggctttttcattt	21.80
t*23*	12345..12368	gcccctatttaaaggggctttttt	11.70
t*29*	complement(16583..16616)	gggagctatcgagaggtagttccctttttagttt	18.20
t*35*	complement(18652..18680)	ggctccttcgggagccttttttattttct	14.90
t*38*	complement(20419..20445)	ggggctgatgcccctttaactatttat	10.90
t*43*	complement(23542..23572)	gggtattaaacacattgtcaatacccttttt	9.20
t*42*	23547..23575	gggtattgacaatgtgtttaatacccttt	10.20
t*64*	41180..41205	gggggagactttaaaaggtcttccccttttttgtttctttt	18.00
t*76*	51080..51100	gggggctgtacagccctcttt	12.50
t*79*	54120..54149	ggctcctttttacgggagcctttttgtttt	12.50
t*80*	complement(54105..54140)	ggctcccgtaaaaaggagccttaaaattttatttt	11.50
t*104*	69298..69323	gccctgtacttagtatggggcttttt	12.20
t*111*	73004..73025	gagcctcttcggaggctctttt	16.10
t*116*	77111..77131	ggtctcttcggagaccttttt	12.80
t*124*	81488..81516	gggaactgtaaaggttccctttttatttt	10.60

**Table 3:** Felix O1 transcription analysis showing the genes maximally expressed early (5 min), delayed early (10 min), middle (25 min) and late (60 min) after infection (pi) of *S*. Typhi with phage Felix O1.

	**TIME (pi)**			
**ORFs maximally expressed**	**5 min**	**10 min**	**25 min**	**60 min**
*13, 16, 20, 21, 22, 31, 45, 46, 48, 49, 54, 58, 62, 69, 70, 71, 74, 75, 79, 95, 105*	+			
*2, 5,9,12, 81*		+		
*1, 3, 4, 6,10,14,17,19, 23, 24, 25, 26, 28, 29, 32, 33, 34, 35, 38, 41, 42, 44, 52, 55, 57, 59, 61, 66, 67, 68, 78, 80, 83, 84, 87, 88, 89, 90, 93, 96, 97, 98, 99, b101, 104, 108, 111, 112, 114, 115, 120, 122, 124, 125, 126, 127, 128, 129, 130*			+	
*30, 36, 37, 51, 53, 63, 64, 72, 73, 76, 77, 106, 107, 109, 117, 121*				+

**Table 4A:** Bacteriophage Felix O1 proteins identified as a result of whole-phage shotgun analysis (WSA) using LTQ-FT LC MS/MS analysis and Mascot search against an in-house database. In order to minimize false positives only data for proteins in which coverage ≥25% are included though this eliminated two likely proteins (orf*76*, tail fiber protein GP37, 9% coverage; and, orf*71*, baseplate protein, 15% coverage). The top 17 proteins are presented (peptide score >40, mass accuracy <2ppm, false discovery rate <0.65).

	**Annotation**	**Mass**	**Score**	**Peptides**	**% Coverage**
gp*77*	tail fiber protein	84006	1310	19	31
gp*73*	conserved protein	53071	1244	22	58
gp*67*	conserved protein	80321	1218	24	40
gp*63*	conserved structural protein	48918	1072	22	41
gp*58*	capsid protein	41580	958	20	48
gp*36*	tail protein	31610	813	14	44
gp*68*	conserved protein	28700	612	10	29
gp*54*	hypothetical protein	18526	498	10	52
gp*74*	hypothetical protein	31597	444	10	42
gp*53*	conserved protein	55525	399	12	25
gp*57*	hypothetical protein	13728	372	8	44
gp*72*	conserved protein	15648	309	5	38
gp*64*	conserved protein	16268	262	5	40
gp*69*	conserved structural protein	13289	211	4	41
gp*55*	hypothetical protein	11715	165	3	30
gp*23*	hypothetical protein	9679	134	4	53
gp*59*	hypothetical protein	17233	119	5	28

**Table 4B:** Peptides, located within the sequence of the major capsid protein of phage Felix O1 indicated in red, which were identified by the WSA procedure.

MLTNSEKSRFFLADLTGEVQSIPNTYGYISNLGLFRSAPITQTTFLMDLTDWDVSLLDAVDRDSRK AETSAPERVR QISFPMMYFKEVESITPDEIQGVRQPGTANELTTEAVVRAKKLMKIRTK FDITREFLFMQALKGKVVDAR GTLYADLYKQFDVEKKTIYFDLDNPNADIDASIEELRMHMEDEAK TGTVINGEEIHVVVDRVFFSKLTKHPKIR DAYLAQQTPLAWQQITGSLRTGGADGVQAHMNTFYYGGVKFVQYNGKFKDKRGKVHTLVSIDSVADTVGVGHAFPNVAMLGEANNIFEVAYCPK MGYANTLGQELYVFEYEKDRDEGIDFEAHSYMLPYCTRPQLLVDVRADAKGG
