# Supplementary material for: Complete Genomic Sequence of Bacteriophage Felix O1
Source: Viruses. 2010 Mar 9;2(3):710–30. doi: 10.3390/v2030710 (PMC3185647; doi:10.3390/v2030710)

Supplementary Tables and Figures for article "Complete Genomic Sequence of Bacteriophage Felix O1", published 9 March 2010.

Supplementary Figure 1. Enhanced physical and genetic map of Felix O1 showing genes with homologs in red, those without in black; promoters ( ) and terminators ( ). The horizontal coloured boxes indicate the function of the genes: green (lysis), grey (HNH endonucleases), pink (morphogenesis) and light blue (DNA replication and nucleotide metabolism).

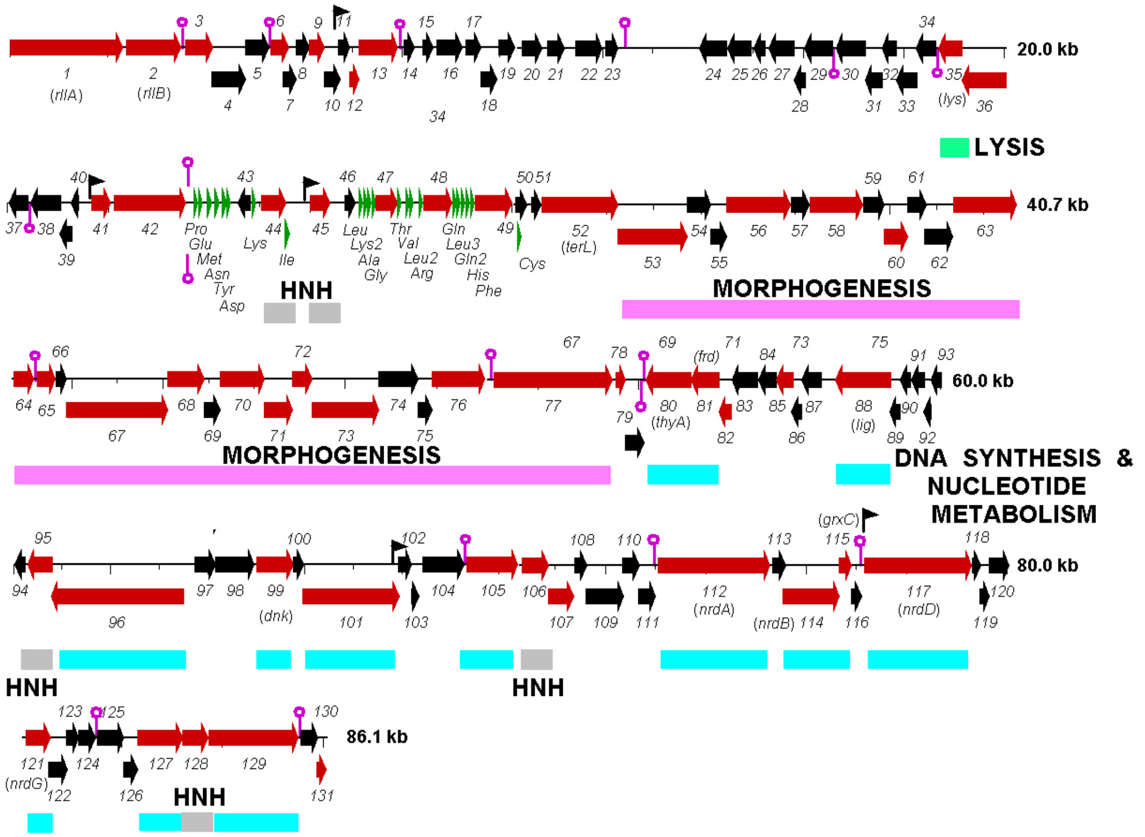

Supplementary Table 1. Characteristics of the proteins encoded by the Felix O1 genome, including physical properties and the presence of homologs other than those for coliphage wV8 and *Erwinia* phage φE a21-4. The RT-PCR results are also indicated for the genes which were examined.

ORF	Product[a]	Positions	Size (aa)/ pI	Homologs[b]	% Identity[c]	RT-PCR[d]
1	rIIA	1–2292	763/7.8	rIIA (NP_899390) [*Vibrio* phage KVP40]	24.4	+
2	rIIB	2372–3481	369/9.1	rIIB (NP_861963) [Enterobacteria phage RB69]	34.0	+
3	CHP	3582–4130	182/9.7	Vs.1 CHP (NP_944162) [*Aeromonas* phage Aeh1]	31.6	+
4		4108–4803	231/5.4	-		+
5		4814–5278	154/9.6	-		+
6	CHP; membrane protein	5331–5678	115/7.0	CHP (YP_001671784) [Enterobacteria phage phiEco32]	32.5	+
7		5572–5841	89/8.5	-		NT
8		5838–6107	89/7.3	-		NT
9		6110–6418	102/4.3	HP 201phi2-1p308 (YP_001957029) [*Pseudomonas* phage 201phi2-1]	32.8	+
10		6408–6734	108/4.7	-		NT
11		6709–6936	75/9.8	-		+
12	CHP	6929–7129	66/4.3	HP rv5_gp026 (YP_002003528) [*Escherichia* phage rv5]	**62.1**	+
13	ADP-ribose binding protein	7130–7915	261/8.5	Tk.4 conserved hypothetical protein YP_003358942[*Deftia* phage φW-14]	23.0	+
14		8042–8254	70/10.1	-		+
15		8423–8629	68/8.7	-		NT
16		8717–9229	170/4.4	-		+
17		9316–9612	98/9.3	-		+
18		9606–9938	110/8.9	-		NT

Supplementary Table 1. *Cont.*

ORF	Product[a]	Positions	Size (aa)/ pI	Homologs[b]	% Identity[c]	RT-PCR[d]
19		9983–10300	105/9.8	-		+
20		10387–10791	134/5.0	-		+
21		10895–11236	113/8.6	-		+
22		11459–11989	176/8.9	-		+
23		12057–12305	82/4.4	-		+
24		13928–14476<	182/6.0	-		+
25		14490–14954<	154/6.5	-		+
26		15008–15232<	74/6.7	-		+
27	Membrane protein	15309–15812<	167/8.1	-		NT
28		15809–16027<	72/9.6	-		+
29		16024–16569<	181/8.2	-		+
30		16642–17223<	193/5.4	-		+
31		17223–17567<	114/9.2	-		+
32		17560–17853<	97/4.3	-		+
33		17853–18248<	131/4.2	-		+
34		18241–18639<	132/5.9	-		+
35	Lysin	18691–19155<	154/9.1	lysozyme (YP_001669615) [*Pseudomonas putida* GB-1]; cd00737, endolysin_autolysin,	**46.1**	+
36	Tail protein	19155–20039<	294/4.5	Major tail subunit (YP_024980) [Vibriophage VP5]	13.4	+
37		20036–20407<	123/9.1	-		+
38		20455–21045<	196/6.1	-		+
39		21039–21272<	77/7.9	-		NT

Supplementary Table 1. *Cont.*

ORF	Product[a]	Positions	Size (aa)/ pI	Homologs[b]	% Identity[c]	RT-PCR[d]
40		21254–21415<	53/9.6	-		NT
41	CHP	21665–22036	123/9.9	HP BamIOP4010DRAFT_2220 (ZP_02890158) [*Burkholderia ambifaria* IOP40-10]	23.6	+
42	CHP	22118–23533	471/8.8	HP KVP40.0300 (NP_899547) [*Vibrio* phage KVP40]	**45.6**	+
43		24573–24803<	76/9.2	-		NT
44	HNH endonuclease	25030–25515	161/9.2	HP phiA1122p09 (NP_848271) [*Yersinia pestis* phage φA1122]	26.3	+
45	HNH endonuclease	26003-26395	130/9.0	HP PSSM2_149 (YP_214381) [Cyanophage P-SSM2]	25.4	+
46		26690–26887	65/10.2	-		+
47	CHP; putative endonuclease VII	27313–27726	137/9.6	HP gp210 (ACH62177) [*Mycobacterium* phage Myrna]	29.1	NT
48	CHP; membrane protein	28262–28819	185/10.1	HP RB43ORF080w (YP_239056) [Enterobacteria phage RB43]	**60.9**	+
49	CHP	29271–30005	244/4.3	HP phiSboM AG3_gp147 (YP_003358634) [*Shigella* phage φSboM-AG3]	26.5	+
50		30069–30296	75/9.6	-		NT
51		30403–30603	66/4.2			+
52	Terminase, large subunit	30625–32226	533/6.0	Terminase large subunit (YP_002003567) [*Escherichia* phage rv5]; pfam03237, Terminase_6	30.6 (C-terminus)	+
53	CHP	32243–33709	488/6.4	HP rv5_gp064 (YP_002003566) [*Escherichia* phage rv5]	27.8	+
54		33709–34209	166/7.8	-		+
55		34209–34541	110/4.3			+

Supplementary Table 1. *Cont.*

ORF	Product[a]	Positions	Size (aa)/ pI	Homologs[b]	% Identity[c]	RT-PCR[d]
56	Head maturation protease (S49 family)	34553–35899	448/4.6	putative head maturation protease (YP_006585) [*Klebsiella* phage φKO2]	25.7	NT
57		35911–36288	125/5.1	-		+
58	CHP; Major capsid protein	36322–37428	368/5.4	CHP ORF26 (NP_758919) [Vibrio phage VHML]	28.8	+
59		37449–37898	149/5.5	-		+
60	CHP	37898–38380	160/10.2	HP VC0395_0791 (YP_001215627) [*Vibrio cholerae* O395]	29.0	NT
61		38377–38778	133/6.0	-		+
62		38753–39352	199/4.6			+
63	CHP; Major structural protein	39353–40705	450/4.8	HP Bpet0987 (YP_001629590) [*Bordetella petrii* DSM 12804] & Structural protein (YP_0020035551) [*Escherichia* phage rv5]	29.7<br><br>27.6	+
64	CHP	40721–41167	148/4.2	Phage protein (YP_001595454) [Enterobacteria phage phiEcoM-GJ1]	27.3	+
65		41241–41639	132/4.8	HP rv5_gp051 (YP_002003553) [*Escherichia* phage rv5]	23.3	NT
66		41642–41881	79/4.4	-		+
67	CHP	41881–44109	742/9.7	HP SSON_1019 (YP_309985) [*Shigella sonnei* Ss046]	30.2	+
68	CHP	44109–44906	265/7.8	Phage conserved protein (ZP_03064971) [*Shigella dysenteriae* 1012]	**69.4**	+
69		44906–45247	113/4.5	-		+
70	CHP	45247–46224	325/8.9	HP Bpet0993 (YP_001629596) [*Bordetella petrii* DSM 12804]	25.1	+

Supplementary Table 1: *Cont.*

ORF	Product[a]	Positions	Size (aa)/ pI	Homologs[b]	% Identity[c]	RT-PCR[d]
71	Putative baseplate protein	46224–46847	207/4.7	putative baseplate protein (YP_003344788) [*Aggregatibacter* phage S1249]	26.6	+
72	CHP	46847–47266	139/5.7	HP rv5_gp044 (YP_002003546) [*Escherichia* phage rv5]	18.1	+
73	CHP	47266–48735	489/4.3	mu-like prophage FluMu protein gp47 (YP_745382) [*Granulibacter bethesdensis* CGDNIH1]<br><br>HP rv5_gp036 (YP_002003538) [*Escherichia* phage rv5]	27.4<br><br>23.9	+
74		48738–49595	285/5.0	-		+
75		49595–49897	100/4.5	-		+
76	Tail fiber	49900–51066	388/7.0	gp37 long tail fiber, distal subunit (NP_049863) [Enterobacteria phage T4]	15.4 (C-terminus)	+
77	Tail fiber	51117–53465	782/8.4	Rac prophage; predicted tail fiber protein (NP_415890) [*Escherichia coli* K12]	22.8 (C-terminus)	+
78	CHP	53545–53739	64/6.6	HP RB49p252 (NP_891823) [Enterobacteria phage RB49]	35.8	+
79	CH membrane protein (1 TMD)	53740–54105	121/8.4	CH membrane P PseT.3 (YP_001595348) [Enterobacteria phage JS98]	24.6	+
80	Thymidylate synthase	54148–55047<	299/5.3	Putative thymidylate synthase (YP_006920) [Bacteriophage T5]; pfam00303, Thymidylat_synt.	39.9	+
81	Dihydrofolate reductase	55049–55594<	181/5.5	Dihydrofolate reductase (YP_002720345) [*Brachyspira hyodysenteriae* WA1]<br>Dihydrofolate reductase (NP_899254) [*Vibrio* phage KVP40]	32.8<br><br>28.3	+

Supplementary Table 1: *Cont.*

ORF	Product[a]	Positions	Size (aa)/ pI	Homologs[b]	% Identity[c]	RT-PCR[d]
82	CHP	55591–55851<	86/6.9	HP RB49p188 (NP_891759) [Enterobacteria phage RB49]	23.9	NT
83		55852–56367<	171/4.6	-		+
84		56381–56740<	119/8.1	-		+
85	Transcriptional regulator	56742–57071<	109/5.3	HP PD1771 (NP_779956) [*Xylella fastidiosa* Temecula1]; pfam01381, HTH_3	19.4	NT
86		57034–57246<	70/5.8	-		NT
87		57248–57631<	127/4.8	-		+
88	DNA ligase	57914–59014<	366/6.8	ATP-dependent DNA ligase (YP_002003586) [*Escherichia* phage rv5]; pfam01068, DNA_ligase_A_M	30.1	+
89		58989–59207<	72/5.7	-		+
90		59204–59410<	68/5.6	-		+
91		59421–59678<	85/8.2	-		NT
92		59668–59814<	48/9.7	-		NT
93		59811–60029<	72/5.6	-		+
94		60026–60235<	69/10.4	-		NT
95	HNH endonuclease	60287–60790<	167/9.6	HNH endonuclease family protein (YP_453589) [*Xanthomonas* phage OP1]	**42.0**	+
96	DNA polymerase	60772–63492<	906/8.2	DNA polymerase1 (NP_775225) [*Pseudomonas aeruginosa* phage PaP3]; pfam00476, DNA_pol_A	18.3 (C-terminus)	+
97		63729–64151	140/7.0	-		+
98		64153–64953	266/4.1	-		+
99	Deoxynucleotide monophosphate kinase	65015–65758	247/8.3	gp1 dNMP kinase (NP_944074) [*Aeromonas* phage Aeh1]	25.7	+

Supplementary Table 1: *Cont.*

ORF	Product[a]	Positions	Size (aa)/ pI	Homologs[b]	% Identity[c]	RT-PCR[d]
100		65767–65967	66/9.2	-		NT
101	Primase/Helicase	65960–67945	661/5.1	DNA primase/helicase (YP_002048644) [*Morganella* phage MmP1]; cd01122, GP4d_helicase	28.3	+
102		67920–68201	93/6.6	-		NT
103		68198–68347	49/10.2	-		NT
104		68420–69277	285/4.3	–		+
105	Exodeoxy-ribonuclease	69340–70386	348/6.2	36L, 5'-exonuclease (NP_858984) [*Xanthomonas oryzae* phage Xp10]	21.1	+
106	HNH endonuclease	70340–70864	174/9.6	RB16 HNH(AP2) 3 (AAY44388) [Enterobacteria phage RB16]	**43.1**	+
107	CHP	70861–71361	166/9.6	hypothetical cyanophage protein (YP_003097284) [*Synechococcus* phage S–RSM4]	28.8	+
108		71383–71631	82/6.1	-		+
109		71607–72362	251/4.7	-		+
110		72343–72666	107/5.8	-		NT
111		72659–72997	112/7.1	-		+
112	NrdA (aerobic ribonucleotide reductase, alpha subunit	73044–75278	744/8.4	Ribonucleoside triphosphate reductase alpha chain (YP_002003607) [*Escherichia* phage rv5]; PRK09103, ribonucleotide-diphosphate reductase subunit alpha	**55.2**	+
113		75334–75591	85/8.9	-		NT
114	NrdB (aerobic ribonucleotide reductase, beta subunit)	75588–76661	357/4.6	Ribonucleoside triphosphate reductase beta chain (YP_002003608) [*Escherichia* phage rv5]; cd01049, Ribonucleotide Reductase, R2/beta subunit	**47.3**	+

Supplementary Table 1: *Cont.*

ORF	Product[a]	Positions	Size (aa)/ pI	Homologs[b]	% Identity[c]	RT-PCR[d]
115	GrxC (glutaredoxin)	76661–76903	80/7.0	putative glutaredoxin (YP_003358567) [*Shigella* phage phiSboM-AG3]	35.0	+
116	Membrane protein (2 TMD)	76896–77102	68/5.1	-		NT
117	NrdD (anaerobic ribonucleotide reductase, alpha subunit)	77151–79295	714/6.4	anaerobic ribonucleoside-triphosphate reductase (ZP_05853341) [*Blautia hansenii* DSM 20583]<br><br>NrdD anaerobic ribonucleotide reductase subunit (YP_239237) [Enterobacteria phage RB43]; PRK09263, anaerobic ribonucleoside triphosphate reductase	**44.9**<br><br>31.0	+
118	Membrane protein (1 TMD)	79310–79474	54/8.0	-		NT
119	Membrane protein (2 TMD)	79450–79650	66/9.3	-		NT
120		79647–80042	131/4.2	-		+
121	NrdG (anaerobic ribonucleotide reductase, beta subunit)	80102–80587	161/6.0	NrdG anaerobic NTP reductase, small subunit (NP_861774) [Enterobacteria phage RB69]	**46.0**	+
122		80550–80927	125/8.1	-		+
123		80894–81151	85/11.3	-		+
124		81154–81474	106/8.2	-		+
125		81526–82041	171/8.3	-		+
126		82034–82312	92/4.9	-		+

Supplementary Table 1: *Cont.*

ORF	Product[a]	Positions	Size (aa)/ pI	Homologs[b]	% Identity[c]	RT-PCR[d]
127	Ribose-phosphate pyrophospho-kinase	82324–83205	293/5.6	HP ESA_04383 (YP_001440398) [*Enterobacter sakazakii* ATCC BAA-894]; COG0462, PrsA, Phosphoribosyl-pyrophosphate synthetase	38.0	+
128	HNH endonuclease	83214–83705	163/9.8	49L HNH endonucleases (NP_858997) [*Xanthomonas oryzae* bacteriophage Xp10]	**40.0**	+
129	Nictotinate phoshoribosy-l transferase	83723–85504	593/5.2	gp133 (YP_001468513) [*Listeria* phage A511]; cd01567, NAPRTase_PncB	34.5	+
130		85559–85894	111/8.8	-		+
131	CHP, Membrane protein (1 TMD)	85876–86052	58/8.0	HP EpJSE_00047 (YP_002922119) [Enterobacteria phage JSE]	39.0	NT

Searches run January 8, 2010; [a]CHP = conserved hypothetical protein; TMD = transmembrane domain; [b]HP = hypothetical protein; [c] %amino acids identity determined using ALIGN. In four cases (C-terminus) = homology largely to C-terminus of the protein; [d]NT = not tested.

Supplementary Table 2. Codon usage statistics for FelixO1 and *Salmonella* species and the utilization of specific amino acids by the phage and its host. *Salmonella* codon usage is an average of *Salmonella enterica* subsp. enterica serovar Choleraesuis, *Salmonella* Typhi Ty2, *Salmonella* Paratyphi A strain ATCC, and *Salmonella* Typhimurium LT2 with data derived from Codon Usage Database (http://www.kazusa.or.jp/codon/). The amino acid utilization is based on an analysis of *Salmonella* choleraesuis.

Amino acid	Codon	Phage fraction	Host fraction	% of total phage amino acids	% of total host amino acids
Gly	GGG	0.09	0.16	5.7	7.4
Gly	GGA	0.15	0.12		
Gly	GGT	0.64	0.24		
Gly	GGC	0.12	0.48		
Glu	GAG	0.37	0.37	5.5	5.6
Glu	GAA	0.63	0.63		
Asp	GAT	0.61	0.61	5.0	5.2
Asp	GAC	0.39	0.39		
Val	GTG	0.12	0.36	6.2	7.1
Val	GTA	0.31	0.16		
Val	GTT	0.44	0.22		
Val	GTC	0.12	0.26		
Ala	GCG	0.04	0.44	5.7	9.8
Ala	GCA	0.42	0.13		
Ala	GCT	0.47	0.13		
Ala	GCC	0.07	0.30		

Supplementary Table 2. *Cont.*

Amino acid	Codon	Phage fraction	Host fraction	% of total phage amino acids	% of total host amino acids
Arg	AGG	0.11	0.03	5.9	5.7
Arg	AGA	0.40	0.04		
Arg	CGG	0.02	0.12		
Arg	CGA	0.08	0.06		
Arg	CGT	0.33	0.33		
Arg	CGC	0.06	0.41		
Ser	AGT	0.20	0.13	7.5	5.8
Ser	AGC	0.11	0.30		
Ser	TCG	0.02	0.165		
Ser	TCA	0.28	0.11		
Ser	TCT	0.32	0.13		
Ser	TCC	0.06	0.17		
Lys	AAG	0.41	0.26	6.6	4.3
Lys	AAA	0.59	0.74		
Asn	AAT	0.54	0.47	5.0	3.8
Asn	AAC	0.46	0.53		
Met	ATG	1.00	1.00	2.0	2.8
Ile	ATA	0.08	0.09	4.9	6.0
Ile	ATT	0.60	0.50		
Ile	ATC	0.32	0.41		
Thr	ACG	0.06	0.34	6.1	5.5
Thr	ACA	0.39	0.105		
Thr	ACT	0.44	0.13		
Thr	ACC	0.11	0.425		

Supplementary Table 2. *Cont.*

**Amino acid**	**Codon**	**Phage fraction**	**Host fraction**	**% of total phage amino acids**	**% of total host amino acids**
Trp	TGG	1.00	1.00	1.7	1.5
Cys	TGT	0.63	0.42	2.8	1.2
Cys	TGC	0.37	0.58		
Tyr	TAT	0.56	0.60	4.3	5.5
Tyr	TAC	0.44	0.40		
Leu	TTG	0.12	0.12	8.1	10.7
Leu	TTA	0.27	0.125		
Leu	CTG	0.14	0.50		
Leu	CTA	0.12	0.05		
Leu	CTT	0.28	0.11		
Leu	CTC	0.07	0.10		
Phe	TTT	0.59	0.60	3.9	3.9
Phe	TTC	0.41	0.40		
Gln	CAG	0.49	0.705	3.4	4.4
Gln	CAA	0.51	0.295		
His	CAT	0.55	0.58	2.1	2.3
His	CAC	0.45	0.42		
Pro	CCG	0.07	0.55	3.1	4.5
Pro	CCA	0.52	0.13		
Pro	CCT	0.35	0.16		
Pro	CCC	0.06	0.165		
End	TGA	0.27	0.31		
End	TAG	0.07	0.10		
End	TAA	0.66	0.59		

Supplementary Table 3. PCR primer pairs used in the RT-PCR experiments to investigate bacteriophage Felix O1 gene expression.

ORF	Upper Primer	Lower Primer
1	AAGGCAGTAGGTGACACAGTTG	TTACCGAAAATTGCCTCTACAGC
2	AAAGAGGGCAAGCTGACTAAAA	GAACGCATCTCAACAGCACCAT
3	TGGTGAGAATAACATGGGTAAA	TTCGCAGCATAATCAAAGTCTCT
4	TGAAGACACCGCAAGAGAAGT	AACGGCAGCATGATTAGCA
5	GTTAAAGAAGGTGCTCCGTATC	ACCGTTGCAGCATCAGTAAAGA
6	AGGTGTCTGGGGAGGAT	CTTGGGAGTTTTTGAATGTA
9	GTACCTAGATAATGGCTGGAAA	TGGAGGTACGGCAACAACT
10	ATCGCAGTAGTAGGAACAAGAA	ATAAATCAACATGCTCAATACT
12	ATGGCTGACTTCTGTAAA	CGTTGTCCTTCGTGGTCTA
13	TGCAGGTATCGGTGGACTTGA	GCTCTTCGACAGGTGGGTAACT
14	CGCTCAGGTCGGGCAAGAAAA	AGGGGGAGTAAGAGGGGGAGA
16	CGGCGTGACTGTTAAAAATCCT	TTCCTCTAAATCATCACCGTCAC
17	CGTCGTGTAGTGATTTGCTTAT	CTTTGCCATTTACAGTTATCTCA
19	GAGGATTTACGGGCTGAAC	AACTTGATGCGCTATTACTACAC
20	ATGGCTATTAATAACCGTGAA	GCCGTCTAGCGTGATACC
21	GAATAAAGGCGAAGATGTAAGC	GAGGATAACCGGATTCACGA
22	ATCAGAAAACGGGGCTTATT	AACAGTATTTACAGGGGCTTTAT
23	GCACTGATAACGCCTTTGA	AGTATAGCCATCCTTGAGAAC
24	GAGTGAATCCGTTGTTGTTATC	AGCAGCTTGTCTTCTCAGTA
25	AGCGGCTATCATACTCAAAT	GTTCTCACTTACTAGGGGATGT
26	CCTTCTTATCGGATTTCTTACTA	AATGAGCAGCCTGTTACTTTG
28	ACTTTCCCTCTAGGTGTTACG	ATCGCAGAAGCCAGAATA
29	ACTGGCGTTGTGTTTCTCTT	GTACAGTTTATTTCCGACCAC

Supplementary Table 3. *Cont.*

ORF	Upper Primer	Lower Primer
30	AATGCACTGCGCTTGTTAC	AATATCAGTGTTTGCCTTGTTG
31	GATTCACCACGCATACCTG	AGACAACTACAACCATCCTAAA
32	GTTGACTTTGTTATTGCCTACCA	GTTGTTTTCCCTATTTGTGACTTC
33	AATACCGTTAAGTTCATCAAA	GTTAGCCGTTACAGTGGAG
34	TGCCTTTCCAGAACAGATTT	TCAAGAGGCGACAAGGTG
35	TTCCACCACTGCATAGCCTCTG	CTGCCGGAATCCCAACAATC
36	CAGTTGCGCCTTCTTTGGTGTA	AATGGAAGAAGGGTGGTGAGGA
37	TAACCTTTGTGATGCGGAGAT	TAAGGTTAAAGGTATTGTTGTT
38	GTTCTTACTTGTTTCTGCGTCTG	GAGGGGTCTTCACTATGTTCAAA
41	GACCAAAAGGCTCTCGTAACA	GTCTGCTGCAATGGCTTTCA
42	TCGAGCCGTAGCAATGTAGAAA	AACCACTTAGCTGCCAAACCTG
44	ACAAGAAGCACCCAAAGAGAAG	TGCATTGTAGCCAGTGAGTAGT
45	ATGAAAGATGGTAAGAGAACT	TGGCAATTTGAACATAGC
46	ATTCGGATGTTGCTTTGGTA	AGGTCTTTGCATAGTCTTGTTTA
48	GATGGCTCTGGAGAAACAAAG	GGATGAATAAAGAATGCCAACA
49	ATTCAGTGGTATTATGGGTGTT	AGGGCAGCTTCTTCTTCA
51	TCTATGGATGCACTTCTTGAT	TGTAGCAGTCTCTTCTTTCTTA
52	TTATGGCCTGCTGCAAAGAAAC	CTCCCAATCAGAAACCATCACA
53	GATGTTCTTGCTATGGGTCAGT	GCGGCTTTGGCTATTTG
54	GACAGTTCATGCCTTTTG	CATATACTTTGCCTTTGAC
55	ACCCCAACGACTATTATTTTACA	CGCATTTATCGTTAGCATCC
57	GGTACAAGCCGGAGAAGAAGC	AGGCCAGTTGGTACGAAATCA
58	TGGCTTGGCAACAGATTACT	TTAGCCACCTTTAGCGTCAG
59	GACCCAGCCAATAACCCTCTT	TCGTATCTGGCAGCATCACC

Supplementary Table 3. *Cont.*

ORF	Upper Primer	Lower Primer
61	GTAGTAAAGCGTAAAGTCTCTG	ATGGGATAATTGCTGTGA
62	TGAAGAAACAGGGGCTACAGT	TTACCCCTTTGGAGTCTACATCT
63	TTGCTTGGGGTAATGCTCA	GCCCCTGCTAAGATACCTG
64	TAATCTACGACCCATCAGG	GTCTAACTTCTCACAACCAAAA
66	CACTGACTGATGCTGCTGTA	CATTTTTCCTGCTCTTCTAA
67	CGCAGGCTTTAGGTCTTGTGATT	CATTGGCCTTTTTGGGTTCGTAA
68	TACTGGAAAGACCGCTACTACA	CCCCTTCTTATTCGGATTCACT
69	ATGGTTCTTGACGCTATCT	AATGCCCTTTCTCTTCC
69	GAACTGGAAAGGCATTACAAAA	CATCAGATAGCGTCAAGAACCA
70	TTTGCAAGGTTACTACAGAAGA	ATGGATTTTGCAACGAACTC
71	CATTTCGGTGACTCTGTGTTA	TGGTGCGTGTGCTCATTA
72	TTTTAAGGGCTGGTGAAG	TGTCTGTTAAAATATCTGTTGTA
73	ACGGCTTTAAACGCTGTA	GTTCTGGCAATAATGTCACC
74	ACAGCGTACACATTAGCAAAAA	TACTTCAGCCATAACACCATAAT
75	GGTAACCAGCTAAAAGTATT	GCTCAGTGGCAAAAGAC
76	CTAACTGGTGGTGGACATACTC	AGCGGTTCTTTGCCATACATA
77	ATTTCACGGGCACTAACACG	ACCTTCGCCATCACTAACAACT
78	TTGCGGGTAAGACAGACA	TACGAACTCATCATCAATAGG
79	ATTTTTAGTGTTATTGGGATGTA	AAGGGTTTTGGTAAGTTTTG
80	CTTCCCTGCGTGTTTGTAGTT	TGGCTTTAGCACCTTGTCAT
81	TTGACTGAATCTTAGCGAAAAT	TGCCGTGGCCTCAACATA
83	TTTACCTCCCTCAGAAGATTACT	TCACATGTGGCGACGAA
84	AAACAGCCTGAACATCTACACC	AAGATTAAGCATACTGAGAAGA
87	GCAAGAACGCGATACTGAT	GATTGTTAAAGGTGTTGCTCTA

Supplementary Table 3. *Cont.*

ORF	Upper Primer	Lower Primer
88	CTCGAATAACTAGCCCAACAGG	TGACGAAGCCCATGATAAGTG
89	ATTTTGTTTGTATAGGATTGTC	ATTCTTCGTGTTGATGGTA
90	CAAACTTATGAATGATGCTCTC	TCTACCGTTATGATGTTGTTC
93	ACTCCAGTACGGTCTCTTG	AGTCTGCGCACATTCTTAT
95	CATGTTATAGCTGCCCTCCAC	ACGCCGTACACTAACACTTCAA
96	CTGGGCTGAGTTTTGGTCTACG	GAGCGGTGTGGTTTGAGTTATGT
97	AAAAGAGGTTGCAAAGTGGTA	TTCAGAACGTGGATAATAGTGC
98	AGTCTGATAACGGTGCTTTTG	TTCGTGGGCTTAATTCTTCTAC
99	CTATTGACACGTTTGGGGTTTA	TGTCACGCTCATATTTTGTAGAA
101	AAGGGGCAATTGACCAAGATAA	AATGTCAACTCCGCAACCAGAT
104	AGAAGACGGTAACAAACTG	GGCTTACAACCAAACTTCTC
105	AAGAGCTTGGCCTGACATTTA	GCAGCCTTTTTACCCATCTTTTC
106	GCACTATGACCCCGAAACA	GTGCAGCCCATCTACCTCTT
107	CTGGTAAGACTCGGAAGAAGA	ATATTGGCATTGTAGAAGAGTC
108	TGACAGCAAGATTGAAGAACT	CATTAGGTGCTGCATTTGAGAT
109	GTTCTTGACGGCTTGATTTACCT	AACGCATGTCTCAGATACTACC
111	GTTAAGCACGGGGACACAGT	AACAACGTAATTACCTCGCATCT
112	AGACCCGCAGATTGAAGATTTA	TACTGCGTGACCAACTGCCATA
114	GGCAATGGGAAGCAGACAC	TAGCCCTTTCAGAATCACTTTAT
115	ATGGGCAAAACCTAAAACCTGT	CCTCTGCCATAAAATTCTGTAGC
117	GGGGTTACTGCGCTTGATGA	TACTGGATTCTGCCGCCTCTAC
120	TAAGATACTCAACATTCCATACG	GCGCAAGTCATTAGCATA
121	TGCTTTAACAGGGAGTCTTG	AATCACCTTGCCACCCATAC
122	CAGTGGAGAGCCGAGTAAGTC	TCCAGAGTCCCATTTCGTATC

Supplementary Table 3. *Cont.*

ORF	Upper Primer	Lower Primer
123	ATTAGGTATGCTCACTGGT	TTATTTAGCCTTCTTATTACG
124	CAGGTTTTATTAGTGATGATGA	CTCTTTTGCTTCTCCAGTAA
125	CCCCTAAGATGACCTACGA	AACCATTACCGCCTTCACT
126	AAGGCGGTAATGGTTGTGAA	GTTACGTTTGTTACTGCCATTG
127	ATGACCCTCTGATTGATTACTTA	GATGTTTTGCTGCTTCTACG
128	GAATACTTTTGCGGAAGATTT	CATGAAGTTCTATTCGTTTTCTA
129	AACATCTTTGGGGGAACTATTA	ACAGGCTGGGTGGTCAA
130	ACTGACGGGTAAGGTTGC	CTGTTTGTTTGGATTGTAGAAG

Supplementary Figure 2. The total ion chromatogram of tryptic Felix O1 protein digest.

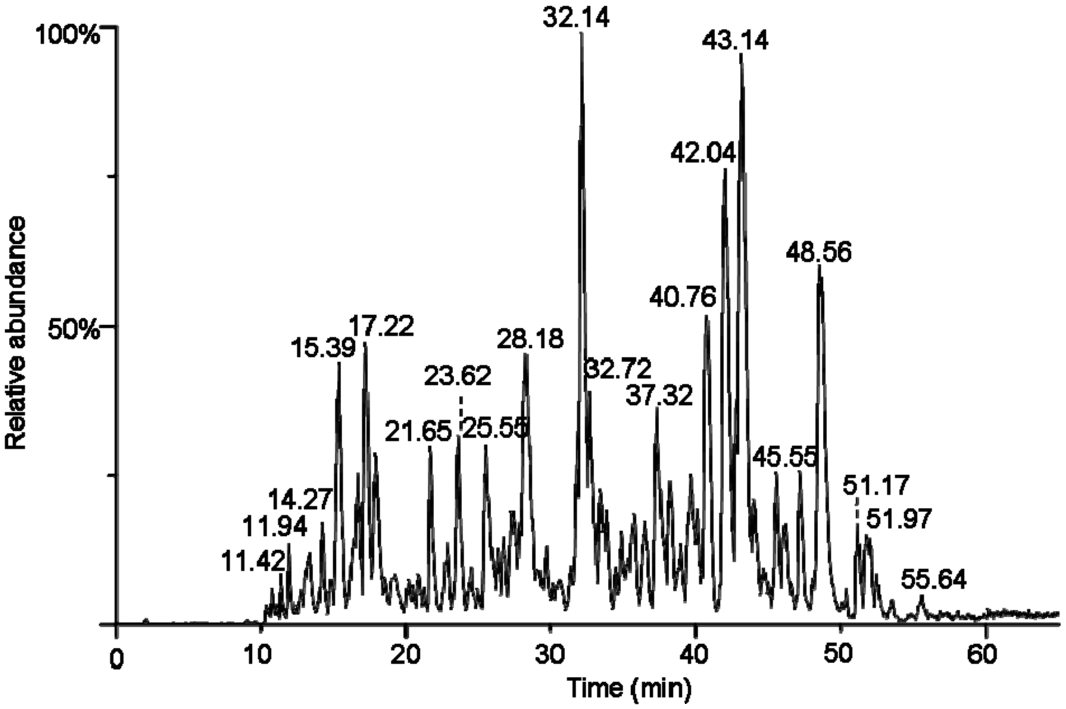

Supplement: Supplementary file 1 [file viruses-02-00710-s001.pdf]
